# Full-Genome Sequence of Human Betacoronavirus 2c Jordan-N3/2012 after Serial Passage in Mammalian Cells

**DOI:** 10.1128/genomeA.00324-14

**Published:** 2014-05-29

**Authors:** Kenneth G. Frey, Cassie L. Redden, Kimberly A. Bishop-Lilly, Reed Johnson, Lisa E. Hensley, Kanakatte Raviprakash, Thomas Luke, Tad Kochel, Vishwesh P. Mokashi, Gabriel N. Defang

**Affiliations:** a Naval Medical Research Center, NMRC-Frederick, Fort Detrick, Maryland, USA; b Henry M. Jackson Foundation, Bethesda, Maryland, USA; c Naval Medical Research Center, Viral and Rickettsial Diseases Department, Silver Spring, Maryland, USA; d National Institutes of Health, National Institute of Allergy and Infectious Diseases, Integrated Research Facility, Fort Detrick, Maryland, USA; e Viral and Zoonotic Diseases Research Program, U.S. Naval Medical Research Unit 3, Abbassia, Cairo, Egypt

## Abstract

Middle East respiratory syndrome coronavirus (MERS-CoV) is the etiologic agent of a highly lethal pneumonia. Here, we report the full-genome sequence of the Jordan-N3/2012 strain after serial passage in two distinct mammalian cell lines. The genome exhibits noteworthy stability, which may inform the development of vaccines and therapeutics used to treat infection with this virus.

## GENOME ANNOUNCEMENT

In October 2012, an unknown betacoronavirus was isolated from a Saudi man with acute pneumonia (1), and the genome sequence of this virus was announced shortly thereafter (2). Reports followed of a similar virus isolated from patients in Qatar, England, and Jordan (3, 4). Subsequently, the virus was designated Middle East respiratory syndrome coronavirus (MERS-CoV) (5). A recent WHO report identified 180 laboratory-confirmed cases of MERS-CoV, including 77 deaths (6). Numerous investigations are being conducted, including efforts to develop vaccines and therapeutics.

It has been reported that MERS-CoV is capable of replicating in a wide variety of mammalian cell types (7). However, it was unclear if serial growth impacts the viral genome. Here, we report the whole-genome sequence of MERS-CoV Jordan-N3/2012 after sequential passage in Vero CCL81 cells and a human embryonic fibroblast line (MRC5). The genome of MERS-CoV is a single-stranded RNA (ssRNA) encoding 10 proteins: a replicase polyprotein, (ORF1ab), three structural proteins (E, N, and M), a surface glycoprotein (S), and five nonstructural proteins (open reading frame [ORF] 3, 4a, 4b, 5, and 8b) (2).

Total RNA was extracted from the supernatants of infected cell cultures, and sequencing libraries were created using the TruSeq RNA sample prep version 2 kit (Illumina, Inc.), beginning the protocol at the fragmentation step. Paired-end 151-base sequencing was performed on the MiSeq (average >730 Mb/sample). The reads were aligned against the NCBI reference (accession no. KC776147.1) using the CLC bio Genomics Workbench (version 6.5). In addition, the sequence reads were *de novo* assembled using CLC bio.

In total, five samples were sequenced: passages 2, 6, 7, and 8 through CCL81 cells and passage 2 through MRC5 cells. All samples shared two single-nucleotide variants (SNVs) compared to the reference strain Jordan-N3/2012. Further analysis of putative SNVs indicated that all samples share an SNV at reference position 24045. This T→C transversion falls in the S gene and results in a nonsynonymous mutation, I→T. This variant is present in 55.8% of the reads in the MRC5 passage and 73.4% of the reads in the CCL81 passage. The proportion of reads with this SNV increased in the Vero-passaged samples, from 34.55% in passage 6 to 93.89% in passage 8, likely representing a cell culture adaptation. This residue is not predicted to reside in the binding domain of the S protein (8). Although the reference genome contains an ambiguous nucleotide (W) at position 11262, all samples show a distinct preference for T (resulting in a leucine versus a histidine residue). It is uncertain as to whether this variation is due to a sequencing error in the reference genome or if population-level differences were lost during cell passage. Although this SNV is present in the coding sequence (CDS) of the ORF1ab gene, the functional consequences are unclear. The residue corresponding to this codon lies between the endopeptidase and the replicase domains and may not be present in mature virions. The apparent stability of this virus *in vitro* may facilitate the development of countermeasures by reducing the potential for rapid evolution and the resulting changes in immunodominant epitopes.

### Nucleotide sequence accession number. 

The genome sequence was deposited in GenBank under the accession no. KJ614529**.**
